# Methyl Jasmonate Applications From Flowering to Ripe Fruit Stages of Strawberry (*Fragaria* × *ananassa* ‘Camarosa’) Reinforce the Fruit Antioxidant Response at Post-harvest

**DOI:** 10.3389/fpls.2020.00538

**Published:** 2020-05-08

**Authors:** Paz E. Zuñiga, Yasna Castañeda, Oscar Arrey-Salas, Lida Fuentes, Felipe Aburto, Carlos R. Figueroa

**Affiliations:** ^1^Institute of Biological Sciences, Campus Talca, Universidad de Talca, Talca, Chile; ^2^Centro Regional de Estudios en Alimentos Saludables, CONICYT-Regional GORE Valparaíso Proyecto R17A10001, Valparaíso, Chile; ^3^Laboratorio de Investigación en Suelos, Aguas y Bosques, Facultad de Ciencias Forestales, Universidad de Concepción, Concepción, Chile

**Keywords:** antioxidants, ascorbate peroxidase (APX), catalase (CAT), flavonoids, *Fragaria* x *ananassa*, preharvest MeJA applications

## Abstract

Preharvest applications of methyl jasmonate (MeJA) have been shown to improve post-harvest fruit quality in strawberry fruit. However, the effectiveness of consecutive field applications at different phenological stages on the reinforcement of the antioxidant capacity remains to be analyzed. To determine the best antioxidant response of strawberry (*Fragaria* × *ananassa* ‘Camarosa’) fruit to different numbers and timing of MeJA applications, we performed three differential preharvest treatments (M1, M2, and M3) consisted of successive field applications of 250 μmol L^–1^ MeJA at flowering (M3), large green (M2 and M3), and ripe fruit stages (M1, M2, and M3). Then, we analyzed their effects on fruit quality parameters [firmness, skin color, soluble solids content/titratable acidity (SSC/TA) ratio, fruit weight at harvest, and weight loss] along with anthocyanin and proanthocyanidin (PA) accumulation; the antioxidant-related enzymatic activity of catalase (CAT), guaiacol peroxidase (POX), and ascorbate peroxidase (APX); the total flavonoid and phenolic contents, antioxidant capacity, and ascorbic acid content (AAC) during post-harvest storage (0, 24, 48, and 72 h). We also evaluated the effect on lignin, total carbon and nitrogen (%C and N), lipid peroxidation, and C and N isotopes signatures on fruits. Remarkably, the results indicated that MeJA treatment increases anthocyanin and PA contents as well as CAT activity in post-harvest storage, depending on the number of preharvest MeJA applications. Also, M3 fruit showed a higher AAC compared to control at 48 and 72 h. Noticeably, the anthocyanin content and CAT activity were more elevated in M3 treatment comparing with control at all post-harvest times. In turn, APX activity was found higher on all MeJA-treated fruit independent of the number of applications. Unlike, MeJA applications did not generate variations on fruit firmness and weight, lignin contents,% C and N, and in lipid peroxidation and water/nitrogen use efficiency according to C and N isotope discrimination. Finally, we concluded that an increasing number of MeJA applications (M3 treatment) improve anthocyanin, PA, AAC, and CAT activity that could play an essential role against reactive oxygen species, which cause stress that affects fruits during post-harvest storage.

## Introduction

Strawberry (*Fragaria* × *ananassa* Duch.), a Rosaceae family member, is one of the most popular fruits grown worldwide due to its organoleptic attributes and abundance in nutrients, vitamins, and minerals (Giampieri et al., 2012; Bertioli, 2019). Since the global living standard increases, the improvement of fruit quality at harvest and to maintain it during storage is a current challenge driven by consumers. In this line, scientific research has been focused on finding preharvest treatments with natural compounds to replace chemical post-harvest treatments due to legal restrictions and the negative perception by consumers (García-Pastor et al., 2020). Strawberries have relevant biological activity in human health due to its important content of bioactive compounds, such as vitamin C (58.8 mg per 100 g fresh weight) and phenolic compounds, including anthocyanins (150 to 600 mg/kg of fresh weight) (reviewed by Giampieri et al., 2012). Also, modification in nutrient and phytochemical composition occurs when the fruit is still attached to the plant and during the development and ripening processes (Alvarez-Suarez et al., 2014). Therefore, the improvement of strawberry fruit quality by preharvest management involves considering physicochemical and functional aspects during the fruit development throughout the season in the field.

The jasmonic acid (JA) and its endogenous plant hormone derived methyl jasmonate (MeJA) have been implicated in several physiological processes, mainly modulating plant defense responses, including antioxidant capacity against pathogens and abiotic stresses (Wasternack, 2007; Wasternack and Strnad, 2016) although they play essential roles in fruit growth and ripening regulation (Concha et al., 2013; Serrano et al., 2018). MeJA is a linolenic acid-derived cyclopentanone-based compound, considered an important plant hormone that can mediate intra- and inter-plant communications due to its ability to diffuse through biological membranes and its volatile nature (Reyes-Díaz et al., 2016). MeJA applied as post- or pre-harvest treatment has been shown to have positive effects on increasing fruit bioactive compounds with antioxidant potential, increasing the beneficial health effects as has been demonstrated in lemon fruit (Serna-Escolano et al., 2019). Nevertheless, the effects of exogenous MeJA application in fruit quality are better known on post-harvest treatments (Reyes-Díaz et al., 2016). For instance, MeJA treatment has been shown to induce plant resistance against the negative impacts of storage (chilling and pathogen attacks injuries), increasing the antioxidant capacity and secondary metabolites content. Less known, the MeJA application as preharvest treatments has several effects, depending on the species, cultivar, edaphoclimatic condition, doses, and phenological stage. In this sense, preharvest MeJA applications have been observed as more effective than post-harvest ones in raspberry cultivars (Flores and Ruiz del Castillo, 2014). It has been reported that preharvest MeJA treatment induces an increase in phenolic and anthocyanin concentrations in different climacteric and non−climacteric fruits during ripening (Martínez-Esplá et al., 2014; Zapata et al., 2014; Ozturk et al., 2015; Flores and Ruiz del Castillo, 2016; García-Pastor et al., 2019; Serna-Escolano et al., 2019). In strawberry, MeJA application has been related to an increase of antioxidant capacity and anthocyanin levels. In *F*. × *ananassa* (‘Aromas’), during an *in vitro* fruit ripening MeJA assay induces an increase in the red coloration of fruit skin along with anthocyanin levels (Garrido-Bigotes et al., 2018). Other research concluded that preharvest applications of 250 μmol L^–1^ MeJA in the Chilean strawberry fruit [*Fragaria chiloensis* (L.) Mill.] increase antioxidant capacity during post-harvest. Moreover, the antioxidant enzymatic related activity also has been associated with MeJA application. In *Arabidopsis thaliana*, the total activities of catalase (CAT), peroxidase (POX), superoxide dismutase (SOD), and glutathione reductase (GR) increased considerably in response to MeJA (Jung, 2004). In this sense, increased activity of the antioxidant enzymes, together with higher levels of antioxidant compounds, as a result of MeJA treatment reinforce would lead to improving and maintaining fruit quality during the post-harvest time. However, the specificity and effectiveness of consecutive field applications at different phenological stages of strawberry fruit on the reinforcement of the antioxidant capacity remain unclear.

In this study, we performed three differential preharvest treatments consisted of successive field applications of 250 μmol L^–1^ MeJA during strawberry fruit development and ripening. Then, we analyzed their effects mainly on fruit quality parameters, anthocyanins and proanthocyanidins (PAs) accumulation, antioxidant enzymatic, and non-enzymatic activity during four post-harvest storage times. This investigation aimed to determine the best combination between the MeJA application frequency and phenological stage of applications to enhance the antioxidant capacity of strawberry (*F.* × *ananassa* ‘Camarosa’) fruit. We found that three MeJA applications from flowering to ripe fruit stages improve the antioxidant response of the fruit during post-harvest storage.

## Materials and Methods

### Plant Material and Treatments

Preharvest field treatments were carried out in a commercial strawberry orchard in Pelluhue, Maule Region, Chile (latitude 35° 47′ S; longitude 72° 33′ W). Climatic conditions of this location are presented in Supplementary Table 1. The experiment was arranged in a randomized complete block design. About 100 plants of *F.* × *ananassa* ‘Camarosa’ distributed in three random plots were used per treatment. Flowers were marked in each plot (*n* = 180) to analyze the same biological material at each developmental stage planned in the experiment. Methyl jasmonate (MeJA) treatments consisted of different sequential applications as sprays on the plant performed through strawberry fruit development. In this sense, M3 treatment consisted in three different applications at flowering, after 24 days at the large green, and after 7 days at 100% red receptacle fruit stages; M2 treatment consisted in two applications at the large green, and after 7 days at 100% red receptacle; and M1 treatment consisted in one application at 100% red stage (Supplementary Figure 1). Each application consisted in 250 μmol L^–1^ MeJA (Sigma-Aldrich, St. Louis, MO, United States) at pH 4.3, and 0.05% (v/v) Tween-20 as surfactant. Distilled water plus 0.05% (v/v) Tween-20 was used as a control (C). The MeJA concentration was chosen as the minimum effective concentration according to previous field experiments on strawberry (Yilmaz et al., 2003; Saavedra et al., 2016, 2017). Harvest was performed at 100% red receptacle after the last MeJA application. Fruits were immediately transported to the laboratory under refrigerated conditions for post-harvest analyses.

### Post-harvest Storage

Four times point evaluations were made at 0, 24, 48, and 72 h during post-harvest storage to check the long-term effect of different preharvest MeJA applications. One hundred eight fruits from each preharvest MeJA treatment were selected for the experiment based on uniform size, shape, and absence of surface damage. For each post-harvest time point, 27 fruits per treatment were separated into three groups of nine fruit each and packaged in transparent perforated plastic containers (24 cm width, 16 cm depth, and 12 cm height) and maintained at room temperature (25°C) and 40% relative humidity.

### Fruit Quality Assessments

Fruit quality measurements were carried out as previously reported (Delgado et al., 2018; Mora et al., 2019). After harvest, all fruits collected from each plot and treatment were weighed (*n* = 352) and expressed as grams per fruit (g⋅Fruit^–1^). Seventeen fruits from each treatment were weighed at each time point (three replicates of nine fruit each) during the post-harvest experiment, and weight loss was expressed as a percentage. Afterward, strawberry skin color from each treatment and post-harvest time point (three replicates of six fruit each) was measured using a colorimeter (model CR-400, Konica Minolta, Tokyo, Japan) and expressed according to the CIELAB scale where L^∗^, a^∗^, and b^∗^ values indicate lightness (dark to light), redness [green (−) to red (+)] and yellowness [blue (−) to yellow (+)], respectively. The dimensions of color Chroma [C = (a^*2^ + b^*2^)^1/2^] and Hue angle [h° = arctan (b^∗^/a^∗^)] were calculated from numerical values of a^∗^ and b^∗^ (McGuire, 1992). Two measurements were taken on opposite sides of each fruit on the equatorial side as technical replicates.

Fruit firmness was measured at opposite sides of 18 fruits from each treatment and post-harvest time point (three replicates of six fruit each) using a texture analyzer (model CT3, Brookfield, MA, United States) fitted with a flat 3 mm TA-39 probe suitable for firmness measurement in berries and small fruits. Each fruit was penetrated 3 mm at 1 mm s^–1^ of speed, and the maximum force was recorded in Newton units (N).

Soluble solids content (SSC) and titratable acidity (TA) analyses were conducted according to Saavedra et al. (2016), with some modifications. Nine fruits of 2 g of fruit receptacle from each treatment and post-harvest time point (three replicates of three fruit each) were homogenized in 5 mL of distilled water employing a controlled speed homogenizer (model MicroDisTec^TM^ HOMOGENIZER 125, Kinematica, Lucerne, Switzerland) with a 12 mm rotor, and filtered. For SSC, 150 μL of each sample was measured using a digital refractometer (model HI 96801, Hanna, Nuşfalǎu, Romania), recorded as Brix degrees (°Bx) and expressed as g of sucrose per 100 g of fresh weight (FW). For TA, aliquots of 2.5 mL of each sample were diluted in distilled water (1:10, v/v) and titrating using semi-automatic titrator (model Digitrate Pro, Jencons Scientific, Ltd., Leighton Buzzard, United Kingdom) with 0.025N NaOH to pH 8.2. Results were calculated as citric acid equivalents (CAE) per 100 g of FW, according to He et al. (2018). Both results were used for the calculation of the SSC/TA ratio.

### Carbon, Nitrogen, and Isotopes Analyses

Lyophilized leaf and fruit tissues were oven-dried to a constant weight, milled, and homogenized in a Spex ball micro mill. An aliquot of the milled samples (six fruits or leaves per treatment) was then weighted with a precision of ± 0.001 mg and encapsulated in tin capsules. Total carbon and nitrogen content (in% of dry weight) and carbon and nitrogen isotope composition (δ^13^C and δ^15^N, respectively, in ‰) were determined using a combustion and gas preparation module (EA-GSL Elemental Analyzer, Sercon, United Kingdom), attached to an Isotope Ratio Mass Spectrometer (20–22 IRMS, Sercon, United Kingdom). Combustion and reduction columns were operated at 1000 and 600°C, respectively, and ultra-grade high purity helium was used as the carrier gas. Combusted gas passed through a GC column operated at 100°C. An ultra-grade reference gas (Ultra High-Grade CO_2_ and N_2_, Indura, Chile) was injected before each analysis for CO_2_ and N_2_ peak drift correction. Two laboratory standards (Corn Flour SCC2256 and Wheat Flour SC2258 Sercon, United Kingdom) were run every 10 analytical samples to ensure analytical quality. These standards were previously calibrated against international reference materials (USGS-40, USGS-41, IAEA-R045, IAEA-600, IAEA-CH-3, IAEA V9, IAEA-C3). The long-term standard deviation of repeated dual-mode δ^15^N and δ^13^C measurements of the laboratory standards were ± 0.2 and ± 0.4‰, for δ^13^C and δ^15^N, respectively.

### Determination of Membrane Lipid Peroxidation

The level of lipid peroxidation in cell membranes was measured in terms of malondialdehyde (MDA), using 2-thiobarbituric acid-reactive substances (TBARS) method described by Cai et al. (2015), with modification. Fruit receptacle (500 mg) from each treatment and post-harvest time point (three replicates of three fruit each) was homogenized with 2 mL 0.1% (w/v) cold trichloroacetic acid (TCA) and centrifuged at 5000 rpm for 20 min at 4°C. Then, a 0.4 mL aliquot of the supernatant fraction was mixed with 1 mL of 20% (w/v) TCA containing 0.67% (w/v) thiobarbituric acid (TBA). The mixture was heated at 100°C for 30 min, quickly cooled in an ice bath, and centrifugated at 15,000 rpm for 15 min. The absorbance of the supernatants was recorded at 532 and 600 nm for the correction of non-specific background absorbance. The MDA concentration was calculated using its molar extinction coefficient (155 mM cm^–1^). The results were expressed as micromoles of MDA per g of FW.

### Antioxidant Capacity, Total Flavonoid Content, and Total Phenolic Content

#### Antioxidant Compounds Extraction

Pooled fruit receptacle (2 g) of each treatment were homogenized in 40 mL of acetone/water/acetic acid (70:29.5:0.5; AWA) solution (Speisky et al., 2012). The resulting mixture was incubated at 30°C for 40 min, with vortexing for 30 s every 10 min, and centrifuged at 5000 rpm for 10 min at 15°C. The supernatant was filtered through a 70 μm nylon cell strainer (Falcon, Corning, NY, United States) and kept at −20°C. The three independent extractions were subjected to the total flavonoid content (TFC), total phenolic content (TPC), and antioxidant capacity determinations.

#### Total Flavonoid Content Determination

Total flavonoid content (TFC) was determined, according to Chang et al. (2002), using quercetin-3-glucoside as the standard. AWA extract from each treatment and post-harvest time point (three replicates of three fruit each) was diluted in methanol:water (1:1) solution and 500 μL of diluted extracts were mixed with 1.5 mL of 95% ethanol, 100 μL of 10% AlCl_3_, 100 μL of 1M CH_3_CO_2_K, and 2.8 mL of distilled water. After incubation at room temperature for 30 min, the OD of the reaction mixture was measured at 415 nm in a UV/Vis spectrophotometer (model V-630, Jasco, Tokyo, Japan), and the results were expressed as mg of quercetin equivalents (QE) per 100 g of FW.

#### Total Phenolic Content Determination

Total phenolic content (TPC) was determined by Singleton and Rossi (1965) method, with modifications suggested by Galati et al. (2003) using gallic acid (GA) as the standard. AWA extract from each treatment and post-harvest time point (three replicates of three fruit each) was diluted in methanol:water (1:1) solution, and 500 μL of the diluted extract was mixed with 3.75 mL of distilled water, 250 μL Folin-Ciocalteu reagent diluted 1:1 in water, and 500 μL of 10% (w/v) sodium carbonate. The mix was homogenized and incubated at room temperature for 1 h. The OD was measured at 760 nm in a UV/Vis spectrophotometer (Model V-630, Jasco), and the results were expressed as mg of GA equivalents (GAE) per 100 g of FW.

#### Antioxidant Capacity Determination

The antioxidant capacity was measured by oxygen-radical absorbing capacity (ORAC) method and assayed for each AWA extract from each treatment and post-harvest time point (three replicates of three fruit each) as described by Speisky et al. (2012) using 2,2′-azobis(2-amidinopropane) dihydrochloride (AAPH) as a source of peroxyl radicals and fluorescein as a source of peroxyl radicals as and the oxidizable probe, respectively. In brief, 20 μL of AWA extract (diluted in 75 mM phosphate buffer, pH 7.4) was transferred to 96-well microplates, each containing 75 μL of APPH (18 mM) and 200 μL of fluorescein (108 nM). The plates were placed measured in a Multi-Mode Microplate Reader (Synergy/HTX, Biotek Instruments, Winooski, VT, United States) and incubated at 37°C for 60 min with shaking every 3 min. During the incubation, the fluorescence was monitored at 485 nm Ex/538 nm Em every 3 min throughout the experiment. The analysis of each sample was performed in triplicate. The results of the ORAC activity were estimated based on a standard curve of Trolox using a quadratic regression equation obtained between the net area under the fluorescence decay curve and the Trolox concentration and the net area under the fluorescence decay curve. ORAC activity was expressed as micromoles of Trolox equivalents (TE) per 100 g of FW.

### Determination of Non-enzymatic Antioxidants

#### Anthocyanin, Proanthocyanidin, and Lignin Contents

Total anthocyanin content (AC) was quantified by the pH differential method (Lee et al., 2005; Debnath and Ricard, 2009), with some modifications (Delgado et al., 2018). Fruit receptacle (2.5 g) was homogenized using 10 mL of absolute ethanol and 1.5N HCl (85:15 v/v) as an extraction solution, incubated overnight at 4°C, and centrifuged for 10 min at 12,000 rpm at 4°C. Two aliquots from the ethanolic phase of each sample were diluted (1:4) with two different buffers: a pH 1 buffer (0.025 M KCl) and a pH 4.5 buffer (0.4 M sodium acetate). Finally, absorbances were quantified at 516 and 700 nm. Extraction solution diluted with each pH buffer were used as blanks. Total anthocyanin content was calculated based on the Lambert-Beer law, using the coefficient of molar extinction for the pelargonidin-3-glucoside (31,620 M^–1^cm^–1^) reported by Swain (1965). Results from each treatment and post-harvest time point (three replicates of three fruit each) were expressed as μg of pelargonidin-3-glucoside equivalent per g of FW.

Total proanthocyanidin content (PA) was quantified by 4-dimethylaminocinnamaldehyde (DMAC) colorimetric method (Prior et al., 2010; Delgado et al., 2018), with some modifications. Fruit receptacle (0.2 g) were ground with liquid nitrogen, homogenized in 1 mL of 80% acetone, and sonicated for 30 min. Aliquots of 70 μL of diluted (1:50) samples were incubated for 20 min with 210 μL of 0.1% DMAC in 80% acidified ethanol and measured at 640 nm using a 96-well microplate reader (model M200, Tecan Trading AG, Switzerland). Ethanol acidified was used as a blank. Total proanthocyanidin content was calculated using linear regression of a calibration curve (0–15.625 μg mL^–1^) of catechin as standard. Results from each treatment and post-harvest time point (three replicates of three fruit each) were expressed as μg of catechin equivalent per g of FW.

Total lignin content was quantified, according to Saavedra et al. (2016). Fruit receptacle (0.25 g) was ground with liquid nitrogen and hydrolyzed as previously described (Meyer et al., 1998; Franke et al., 2002). Extractable cell wall complexes were obtained by the thioglycolic acid (TGA) method (Campbell and Ellis, 1992). The insoluble lignin pellet was dissolved in 1 mL of 1N NaOH, and UV-absorbance was measured at 280 nm using a 96-well plate reader (model M200, Tecan Trading AG). Total lignin content was calculated using linear regression of a calibration curve (0–20 μg mL^–1^) of hydrolytic lignin (Sigma-Aldrich) as standard. Results from each treatment and 0, 24, and 48 h post-harvest time point (three replicates of three fruit each) were expressed as μg of lignin per g of FW.

#### Ascorbic Acid Content

Ascorbic acid content (AAC) was quantified using the 2,6-dichloroindophenol titrimetric method, according to AOAC method 967.21 (Horwitz, 2002; Fuentes et al., 2016). Fruit extracts were prepared from 2 g frozen samples from each treatment and post-harvest time point (three replicates of one fruit each) using 4 mL 3% metaphosphoric acid, homogenized, and centrifuged at 5000 rpm for 15 min. The supernatant was diluted first to 10 ml with 3% metaphosphoric acid and then to 100 mL with ultrapure water. AAC determination was performed through a calibration curve of L-ascorbic acid (Sigma–Aldrich). Measurements were performed four times, and results were expressed as mg of ascorbic acid per 100 g of FW.

### Determination of Antioxidant-Related Enzymatic Activities

The determination of enzyme activities was performed through spectrophotometric activity assays at 25°C. Fruit receptacle (1 g) from each treatment at each post-harvest time point (three replicates of three fruit each) were ground with liquid nitrogen and homogenized in 5 mL of extraction buffer containing 100 mM potassium phosphate buffer (pH 7.8), 1 mM EDTA (pH 7.0) and 5% (w/w) polyvinylpolypyrrolidone (PVPP). Then, the homogenate was centrifuged for 15 min at 10,000 rpm at 4°C. The supernatant was taken as a crude enzyme extract and was used for estimated enzymatic activities, as described below.

#### Catalase (CAT, EC 1.11.1.6)

The CAT activity was determined following the hydrogen peroxide (H_2_O_2_, ε = 36 mM^–1^ cm^–1^) breakdown at 240 nm, as previously described (Garcıìa-Limones et al., 2002; García-Limones et al., 2009). The reaction mixture contained 30 μL 500 mM potassium phosphate buffer (pH 7.0) and 40 μL of crude fruit protein extract in a 232 μL-volume. The reaction started by adding 68 μL of 88 mM H_2_O_2_. Results were expressed as mmoles of H_2_O_2_ decomposed per g of FW.

#### Guaiacol Peroxidase (POX, EC 1.11.1.7)

The POX activity was determined through the oxidation of guaiacol by measure the rate of tetraguaiacol formation at 470 nm (ε = 26,600 mM^–1^ cm^–1^) (Rao et al., 1996). The assay mixture contained 60 μL 500 mM potassium phosphate buffer (pH 7.0), 2.5 μL 10 mM EDTA (pH 7.0), 16 mM guaiacol, and 16.2 μL 88 mM H_2_O_2_, in a 195 μL-volume. The reaction was initiated by adding 105 μL of crude fruit protein extract. Results were expressed as mmoles of tetraguaiacol per g of FW.

#### Ascorbate Peroxidase (APX, EC 1.11.1.11)

The APX activity was determined following the H_2_O_2_-dependent oxidation of ascorbate by measure the rate of dehydroascorbate (DHA) formation at 290 nm (ε = 2.8 mM^–1^ cm^–1^) (Garcıìa-Limones et al., 2002; García-Limones et al., 2009) with a few modifications. The reaction mixture contained 60 μL 500 mM potassium phosphate buffer (pH 7.0), 20 μL 5 mM L-ascorbic acid (Sigma-Aldrich), 20 μL 10 mM EDTA (pH 7.0), and 20 mL of crude fruit protein extract in a 300 μL-volume. The reaction started by adding 1.2 μL 88 mM H_2_O_2_. Results were expressed as mmoles of DHA per g of FW.

### Statistical Analysis

The experiment was performed using a randomized complete block design. All the measurements were conducted at triplicate and expressed as mean ± standard deviation (SD) (tables) or ± standard error of the mean (SEM) (figures). Data were analyzed by analysis of variance (ANOVA) using Infostat software (version 2016) (Rienzo et al., 2016). Tukey *post hoc* test was used to evaluate the significance between treatments at each post-harvest time point (0, 24, 48, and 72 h). Values of *p* ≤ 0.05 were considered statistically significant. Principal component analysis (PCA) was used for all variables to discriminate between treatments and post-harvest times points, using the R software with the ggplot2 package (Wickham, 2016).

## Results

### Fruit Quality Assessments

**FIGURE 1:**
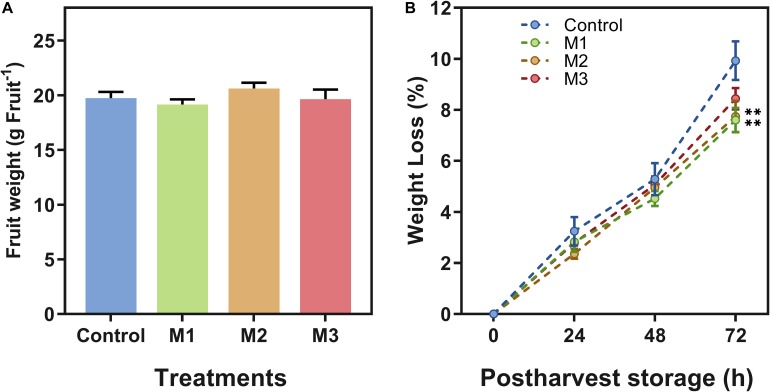
Changes in weight of preharvest methyl jasmonate (MeJA) treated strawberry (*Fragaria* × *ananassa* ‘Camarosa’) fruit at post-harvest. Effect of different MeJA treatments (M1, M2, and M3) or water (control) on strawberry **(A)** fruit weight (g⋅Fruit^–1^) at harvest (0 h post-harvest), and **(B)** fruit weight loss (%) during post-harvest storage (0, 24, 48, 72 h). Data represent mean ± SEM (*n* = 352 for fruit weight; *n* = 27 for fruit weight loss). Differences between treatments were determined by ANOVA and Tukey test. Asterisks indicate significant differences (**p ≤ 0.01).

Different field MeJA applications seem to have little or no impact on fruit firmness and weight attributes. Regarding that, no differences between treatments on strawberry (*F.* × *ananassa* ‘Camarosa’) firmness (Table 1) or fruit weight at harvest (Figure 1A) were observed. Fruit weight loss increased during post-harvest in all treatments (Figure 1B), but results showed a significant difference at 72 h on M1 and M2 treatments declining fruit weight loss compared to control fruits (*p* ≤ 0.05).

**TABLE 1:** Changes in firmness, soluble solids content/titratable acidity (SSC/TA) ratio, and color parameters during post-harvest storage (0, 24, 48, and 72 h) of strawberry (*Fragaria* × *ananassa* ‘Camarosa’) fruits treated with three different sequential applications of 250 μmol L^–1^ methyl jasmonate (M1, M2, and M3) or water (control) during preharvest.

Post-harvest storage (h)	Treatment	Firmness (N)	SSC/TA ratio	Color parameters				
				L*	a*	b*	Chroma	Hue angle (h°)
0	Control	0.26 ± 0.10^Aa^	12.76 ± 1.70^Ac^	28.92 ± 1.83^Aa^	27.59 ± 2.74^Ab^	15.13 ± 2.80^Ab^	31.53 ± 3.38^Cb^	28.62 ± 3.53^Aab^
	M1	0.28 ± 0.12^Aa^	12.35 ± 1.41^Ab^	29.22 ± 1.95^Ab^	28.40 ± 2.47^Ab^	14.99 ± 2.98^Aa^	32.18 ± 3.23^ACb^	27.62 ± 3.79^Aa^
	M2	0.36 ± 0.17^Aa^	13.97 ± 1.30^Bb^	30.97 ± 2.38^Bc^	30.74 ± 2.19^Bb^	16.43 ± 3.28^Ab^	34.93 ± 3.15^Bb^	27.90 ± 3.89^Aa^
	M3	0.34 ± 0.15^Aa^	12.60 ± 2.66^Abc^	30.15 ± 2.33^ABc^	30.23 ± 2.43^Bc^	16.29 ± 3.19^Ac^	34.40 ± 3.38^ABc^	28.10 ± 3.62^Ab^
24	Control	0.47 ± 0.20^Ab^	10.19 ± 2.26^Aab^	28.60 ± 1.96^Ba^	24.43 ± 2.88^Aa^	12.34 ± 2.52^Aa^	27.61 ± 3.26^ABa^	26.46 ± 3.95^ABab^
	M1	0.47 ± 0.15^Ab^	11.83 ± 1.18^ABb^	27.24 ± 2.39^ABa^	24.63 ± 2.78^ABa^	13.06 ± 2.84^Aa^	27.74 ± 3.73^ABa^	27.90 ± 3.16^Ba^
	M2	0.41 ± 0.13^Aa^	10.93 ± 1.92^Aa^	28.03 ± 1.76^ABa^	24.05 ± 3.26^Aa^	13.34 ± 2.30^Aa^	29.77 ± 2.98^Ba^	26.51 ± 2.77^ABa^
	M3	0.38 ± 0.13^Aa^	13.37 ± 3.02^Bc^	26.69 ± 1.69^Aa^	26.58 ± 2.39^Ba^	11.26 ± 2.77^Aa^	26.60 ± 3.92^Aa^	24.84 ± 3.56^Aa^
48	Control	0.41 ± 0.18^Ab^	11.40 ± 1.87^Ab^	30.23 ± 2.60^Aa^	26.82 ± 3.08^Aab^	14.99 ± 3.02^Ab^	30.77 ± 3.96^Ab^	28.99 ± 3.11^Ab^
	M1	0.44 ± 0.19^Ab^	12.90 ± 3.15^ABb^	29.44 ± 2.39^Ab^	26.51 ± 3.45^Aab^	14.03 ± 3.53^Aa^	30.08 ± 4.28^Aab^	27.61 ± 4.69^Aa^
	M2	0.44 ± 0.21^Aa^	13.14 ± 1.47^Bb^	29.93 ± 2.32^Abc^	28.21 ± 2.99^Aa^	14.98 ± 3.09^Aab^	32.01 ± 3.72^Aa^	27.81 ± 3.90^Aa^
	M3	0.43 ± 0.17^Aa^	11.62 ± 0.88^Aab^	29.58 ± 2.36^Abc^	28.40 ± 3.51^Abc^	14.03 ± 3.28^Ab^	31.73 ± 4.39^Abc^	26.03 ± 3.51^Aab^
72	Control	0.43 ± 0.13^Ab^	9.71 ± 0.52^Ba^	29.25 ± 2.55^Aa^	27.38 ± 3.69^Ab^	13.60 ± 3.43^Aab^	30.63 ± 4.63^Ab^	26.11 ± 3.65^Aa^
	M1	0.39 ± 0.17^Aab^	8.72 ± 0.87^Aa^	29.55 ± 2.00^Ab^	27.50 ± 2.80^Ab^	13.47 ± 2.97^Aa^	30.69 ± 3.52^Ab^	25.90 ± 3.81^Aa^
	M2	0.41 ± 0.12^Aa^	10.27 ± 1.38^Ba^	28.87 ± 2.16^Aab^	27.42 ± 3.94^Aa^	13.15 ± 3.26^Aa^	30.54 ± 4.18^Aa^	25.60 ± 5.95^Aa^
	M3	0.44 ± 0.12^Aa^	10.29 ± 2.34^Ba^	28.54 ± 2.25^Ab^	26.78 ± 3.06^Ab^	12.39 ± 2.89^Aab^	29.58 ± 3.64^Ab^	24.63 ± 4.03^Aa^

*Data show mean values ± SD of three replicates (three fruits by replicate for SSC/TA ratio, and six fruits by replicate for firmness and color parameters). For each parameter, different capital letters indicate a significant difference between treatments within each post-harvest time point, and different lower-case letters indicate significant differences of each treatment between time points during post-harvest (p ≤ 0.05). For experimental details (see section “Materials and Methods”).*

The SSC/TA ratio gradually decreased during post-harvest in all treatments, but higher values of SCC/TA ratio were found in MeJA-treated fruits at 0 (M2), 24 (M3), and 48 h (M2) compared with control, which means that MeJA treatments impact this important parameter in strawberry flavor by increasing SSC and reducing TA values (Table 1 and Supplementary Table 2). In turn, differences in fruit color were found at 0 and 24 h between MeJA-treated and control fruits (Table 1). In this sense, significant differences in luminosity (L^∗^ index) were observed between control (28.92 ± 1.83) and M2 (30.97 ± 2.38) treatments at 0 h and a constant decrease from 0 to 72 h during post-harvest storage in M2 and M3 treatments. We observed an increase in redness (a^∗^ index) in M2 (30.74 ± 2.19) and M3 (30.23 ± 2.43) treatments at 0 h compared with control (27.59 ± 2.74), although this index decrease through storage time in those treatments. At 24 h of storage, results showed significant differences between control (28.60 ± 1.96) and M3 treatment (26.69 ± 1.69) in luminosity, and between control (24.43 ± 2.88) and M3 treatment (26.58 ± 2.39) in redness. No significant differences were observed at 48 and 72 h of post-harvest storage in any of the color parameters analyzed. The results on fruit skin luminosity and redness indicate that MeJA treatments could increase luminosity (whiter) and redness in fruits at 0 h, although this effect is loss from 24 to 72 h post-harvest storage (Table 1).

**FIGURE 2:**
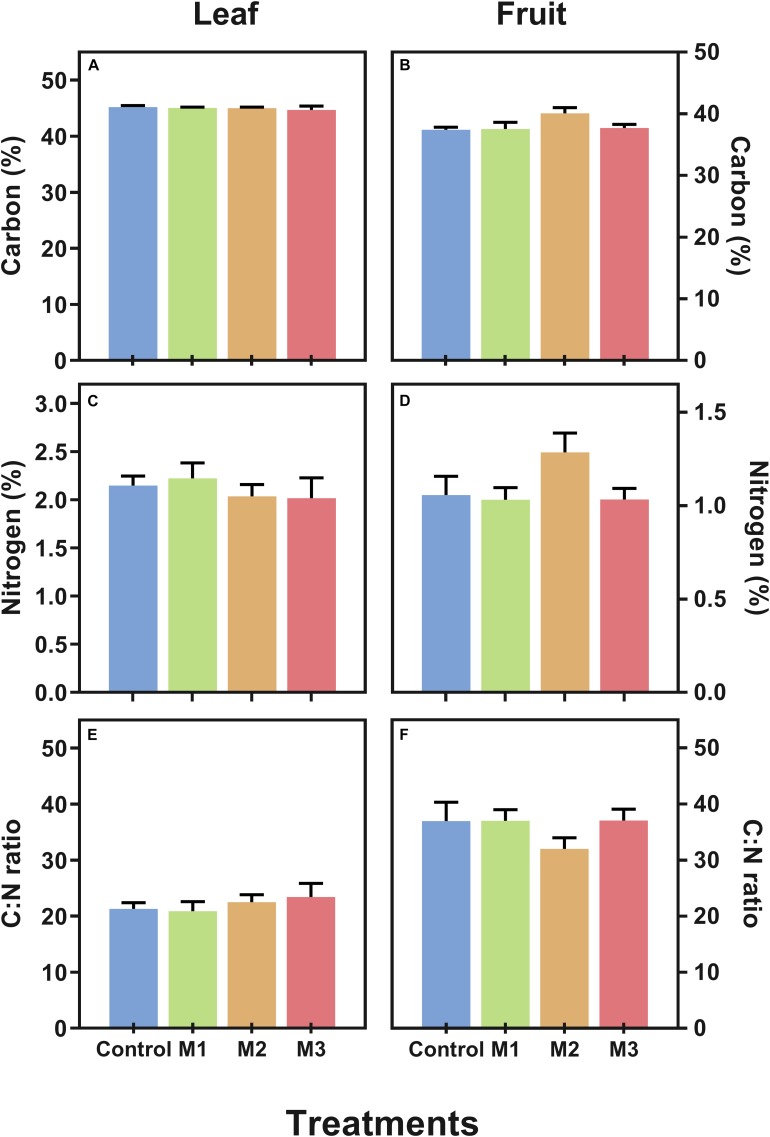
Changes in carbon (C) and nitrogen (N) contents of preharvest methyl jasmonate (MeJA) treated strawberry (*Fragaria* × *ananassa* ‘Camarosa’) leaf and fruit at harvest. Effect of different MeJA treatments (M1, M2, and M3) or water (control) on **(A)** leaf carbon content (%), **(B)** fruit carbon content (%), **(C)** leaf nitrogen content (%), **(D)** fruit nitrogen content (%), **(E)** leaf C:N ratio, and **(F)** fruit C:N ratio of MeJA-treated strawberry plants at harvest.% of dry weight. Data represent mean ± SEM (*n* = 6).

Additionally, with the aim to verify if MeJA alters essential constituents of plants we measured the carbon (C) and nitrogen (N) contents (as% of dry weight) and estimated the C:N ratio in leaf (Figures 2A,C,E) and fruit (Figures 2B,D,F) of MeJA-treated strawberries at harvest. No alterations in C, N, and C:N ratio were observed both in leaf and fruit tissues, which could imply no changes in main metabolic pathways as results of MeJA preharvest applications.

### Isotope Analyses and Lipid Peroxidation Determination

**FIGURE 3:**
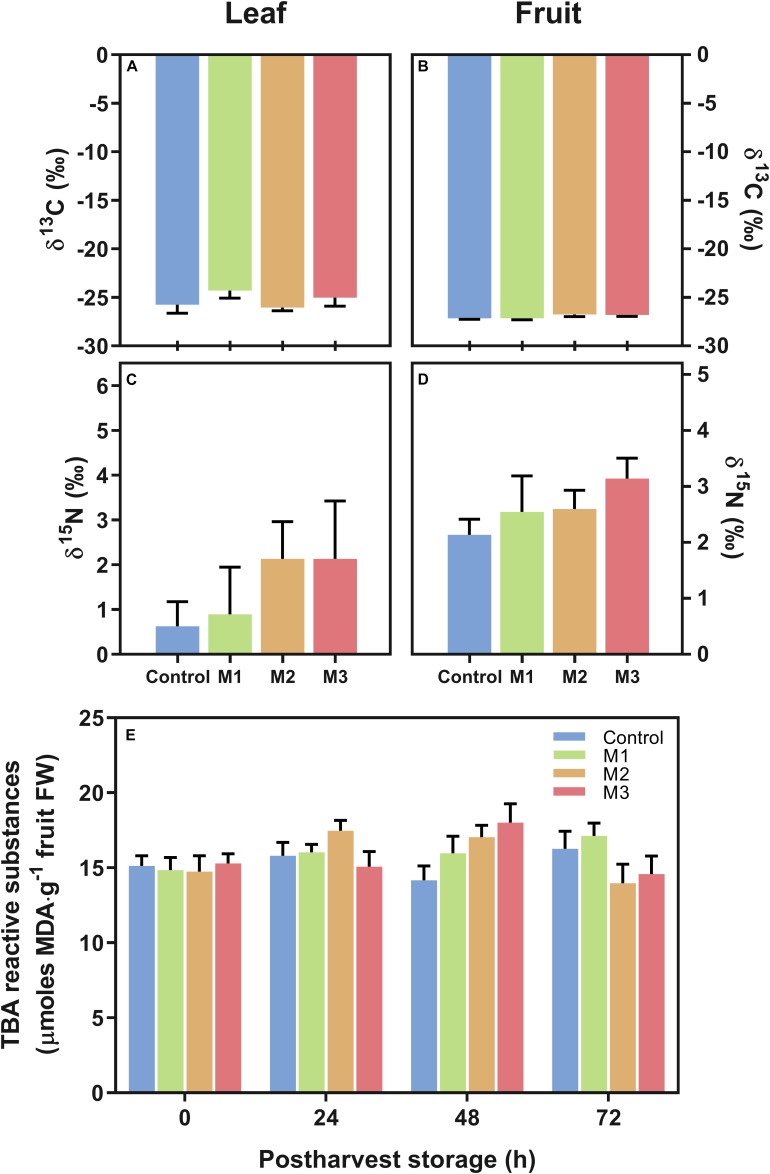
Changes in carbon (C) and nitrogen (N) corresponding isotopes (δ^13^C and δ^15^N) and in malondialdehyde (MDA) content of preharvest methyl jasmonate (MeJA) treated strawberry (*Fragaria* × *ananassa* ‘Camarosa’) samples. Effect of different MeJA treatments (M1, M2, and M3) or water (control) on **(A)** leaf δ^13^C (‰), **(B)** fruit δ^13^C (‰), **(C)** leaf δ^15^N (‰), **(D)** fruit δ^15^N (‰), and **(E)** fruit lipid peroxidation during post-harvest storage (μmoles MDA per g of fresh weight). Data represent mean ± SEM (*n* = 6) and mean± SEM (*n* = 9) for isotope and lipid peroxidation analyses, respectively.

In the present study, we study the changes in C and N isotopes (δ^13^C and δ^15^N) in leaf and fruit at harvest and in lipid peroxidation during post-harvest fruit storage as measurements indicative of stress in different MeJA-treated samples. No differences were found in δ^13^C and δ^15^N stable isotope composition, used as a proxy for water use efficiency (WUE) and differential nutrient source/allocation, respectively, both in fruits or leaves (Figures 3A–D). Additionally, there were no significant differences in strawberry fruit lipid peroxidation (expressed total MDA) during post-harvest storage between treatments (Figure 3E). These results suggest that MeJA treatments do not alter the stress status of leaf and fruit at harvest and fruits at post-harvest storage in comparison to control samples.

### Determination of Non-enzymatic Antioxidants

#### Total Flavonoid Content, Total Phenolic Content, and Antioxidant Capacity

The TFC, TPC, and antioxidant capacity of preharvest MeJA treatment fruit during post-harvest storage are shown in Table 2. In the present work, no differences were found between control and MeJA treatments on antioxidant capacity and no variation was observed through post-harvest storage in all treatments. Alongside that, no significant differences were found on TFC, where values were spread from 5.4 ± 0.9 to 11.2 ± 1.0 for M3-treated, and from 6.7 ± 1.7 to 10.4 ± 0.7 for control fruits. The TPC values showed no significant changes between treatments either with 157.87 ± 32.01 and 146.22 ± 4.74 mg GAE⋅100 g^–1^ FW for control and M3 treatment at 0 h, respectively. However, we noted a significant decrease of TFC in MeJA treatments from 24 to 48 h (M1 and M3) and from 0 to 48 h (M2) of post-harvest storage (Table 2).

**TABLE 2:** Changes in total flavonoid content (TFC), total phenolic content (TPC), and antioxidant capacity determined by Oxygen Radical Absorbance Capacity (ORAC) during post-harvest storage (0, 24, 48, and 72 h) of strawberry fruits treated with three different sequential applications of 250 μmol L^–1^ methyl jasmonate (M1, M2, and M3) or water (control) during preharvest.

Post-harvest storage (h)	Treatments	TFC (mg QE ⋅100 g^–1^ FW)	TPC (mg GAE ⋅100 g^–1^ FW)	Antioxidant capacity (μmoles TE 100 g^–1^ FW)
0	Control	8.76 ± 2.36^Aa^	157.87 ± 32.01^Aa^	3902.67 ± 214.65^Aa^
	M1	8.76 ± 0.68^Abc^	138.41 ± 8.01^Aa^	4340.00 ± 2203.34^Aa^
	M2	9.94 ± 1.76^Ab^	133.73 ± 8.90^Aa^	4309.67 ± 819.29^Aa^
	M3	10.79 ± 0.56^Ab^	146.22 ± 4.74^Aab^	4768.67 ± 468.84^Aa^
24	Control	10.41 ± 0.65^Aa^	142.98 ± 7.15^Aa^	3866.50 ± 1091.07^Aa^
	M1	9.64 ± 1.39^Ac^	137.94 ± 8.29^Aa^	4341.67 ± 683.73^Aa^
	M2	9.41 ± 1.33^Aab^	131.77 ± 33.85^Aa^	3544.33 ± 1032.03^Aa^
	M3	11.24 ± 0.99^Ab^	132.13 ± 12.43^Aa^	3913.67 ± 629.51^Aa^
48	Control	6.72 ± 1.66^Aa^	152.23 ± 4.44^Aa^	3405.00 ± 758.08^Aa^
	M1	6.12 ± 0.87^Aa^	155.61 ± 14.80^Aa^	3878.67 ± 896.53^Aa^
	M2	6.17 ± 1.28^Aa^	161.17 ± 20.84^Aa^	4010.00 ± 1059.49^Aa^
	M3	6.56 ± 0.52^Aa^	157.54 ± 17.76^Aab^	4243.67 ± 1067.63^Aa^
72	Control	7.17 ± 0.26^Aa^	171.92 ± 7.04^Aa^	5086.00 ± 960.98^Aa^
	M1	6.61 ± 0.88^Aab^	166.88 ± 11.46^Aa^	4021.00 ± 210.72^Aa^
	M2	7.02 ± 0.21^Aab^	167.74 ± 9.22^Aa^	2614.33 ± 808.62^Aa^
	M3	5.37 ± 0.89^Aa^	161.96 ± 3.06^Ab^	3174.67 ± 1591.72^Aa^

*Data show mean values ± SD of three replicates (three fruits by replicate). For each parameter, different capital letters indicate a significant difference between treatments within each post-harvest time point, and different lower-case letters indicate significant differences of each treatment between time points during post-harvest (p ≤ 0.05). FW, fresh weight; QE, quercetin equivalents; GAE, gallic acid equivalent; TE, Trolox equivalent. For experimental details (see section “Materials and Methods”).*

#### Anthocyanin, Proanthocyanidin, and Lignin Contents

**FIGURE 4:**
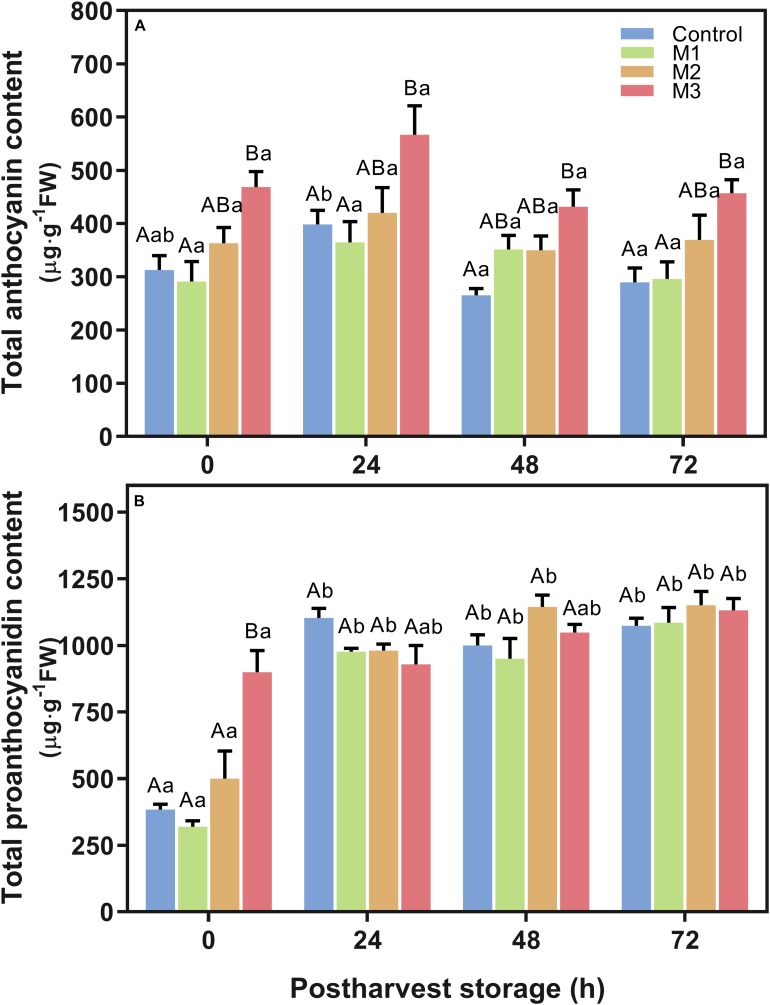
Changes in total anthocyanin and proanthocyanidin contents of preharvest methyl jasmonate (MeJA) treated strawberry (*Fragaria* × *ananassa* ‘Camarosa’) fruit at post-harvest. Effect of different MeJA treatments (M1, M2, and M3) or water (control) on strawberry **(A)** total anthocyanin content (μg pelargonidin-3-glucoside equivalent per g of FW), and **(B)** total proanthocyanidin content (μg catechin equivalent per g of FW) during post-harvest storage. Data represent mean ± SEM (*n* = 9). Differences between treatments were determined by ANOVA and Tukey test. Different capital letters indicate a significant difference between treatments within each post-harvest time point, and different lower-case letters indicate significant differences of each treatment between time points during post-harvest (*p* ≤ 0.05).

Results indicated an increment in total anthocyanin content (Figure 4A) in M3 MeJA-treated fruits respect to control at all time points during post-harvest. The highest anthocyanin content showed by M3 treatment at 24 h, supports the higher a^∗^ index observed in MeJA-treated fruits at that time point (Table 1), unlike that observed at 0 h. None of the MeJA-treated fruits decreases their anthocyanin content during post-harvest storage; contrary to what happened with the control fruits (Figure 4A). Otherwise, differences in total proanthocyanidin content were only noted at 0 h (Figure 4B), being M3-treated fruits, which presented higher levels than those exhibited by the other treatments. All treatments increase their proanthocyanidin contents up to the end of post-harvest storage (Figure 4B). Additionally, no differences were found related to lignin content between MeJA and control treatments during post-harvest storage (Supplementary Figure 2).

#### Ascorbic Acid Content

**FIGURE 5:**
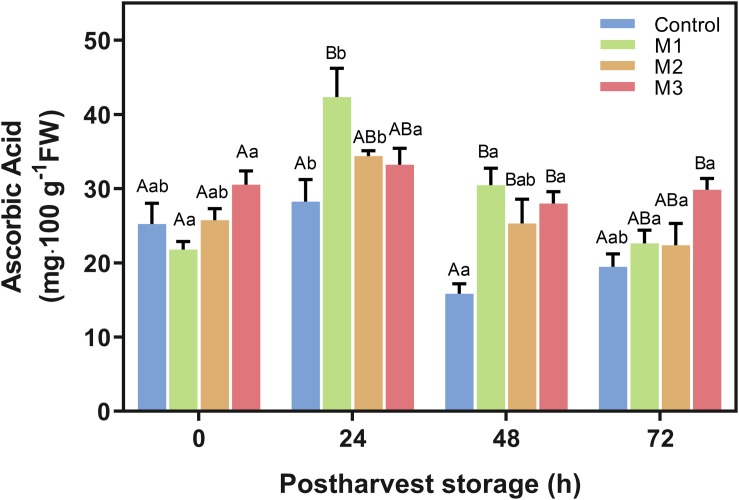
Changes in ascorbic acid content of preharvest methyl jasmonate (MeJA) treated strawberry (*Fragaria* × *ananassa* ‘Camarosa’) fruit at post-harvest. Effect of different MeJA treatments (M1, M2, and M3) or water (control) on strawberry ascorbic acid content (AAC) during post-harvest storage. Data represent mean ± SEM (*n* = 3). Differences between treatments were determined by ANOVA and Tukey test. Different capital letters indicate a significant difference between treatments within each post-harvest time point, and different lower-case letters indicate significant differences of each treatment between time points during post-harvest (*p* ≤ 0.05).

The AAC is shown in Figure 5. M1-treated fruits had markedly higher values than the control treatment at 24 h, but an increase of AAC was observed for all MeJA treated fruits at 48 h. Remarkably, AAC on M3-treated strawberries was higher than control at 48 h (*p* = 0.0016) and remained higher at 72 h (*p* = 0.0092) with an increase of 63.8 and 53.3%, respectively. However, no significant difference was found for M1 and M2 treatments concerning control at 72 h. Moreover, AAC values of M2 and M3 treatments remain stable during storage time points (Figure 5).

### Determination of Antioxidant Enzymatic Activities

**FIGURE 6:**
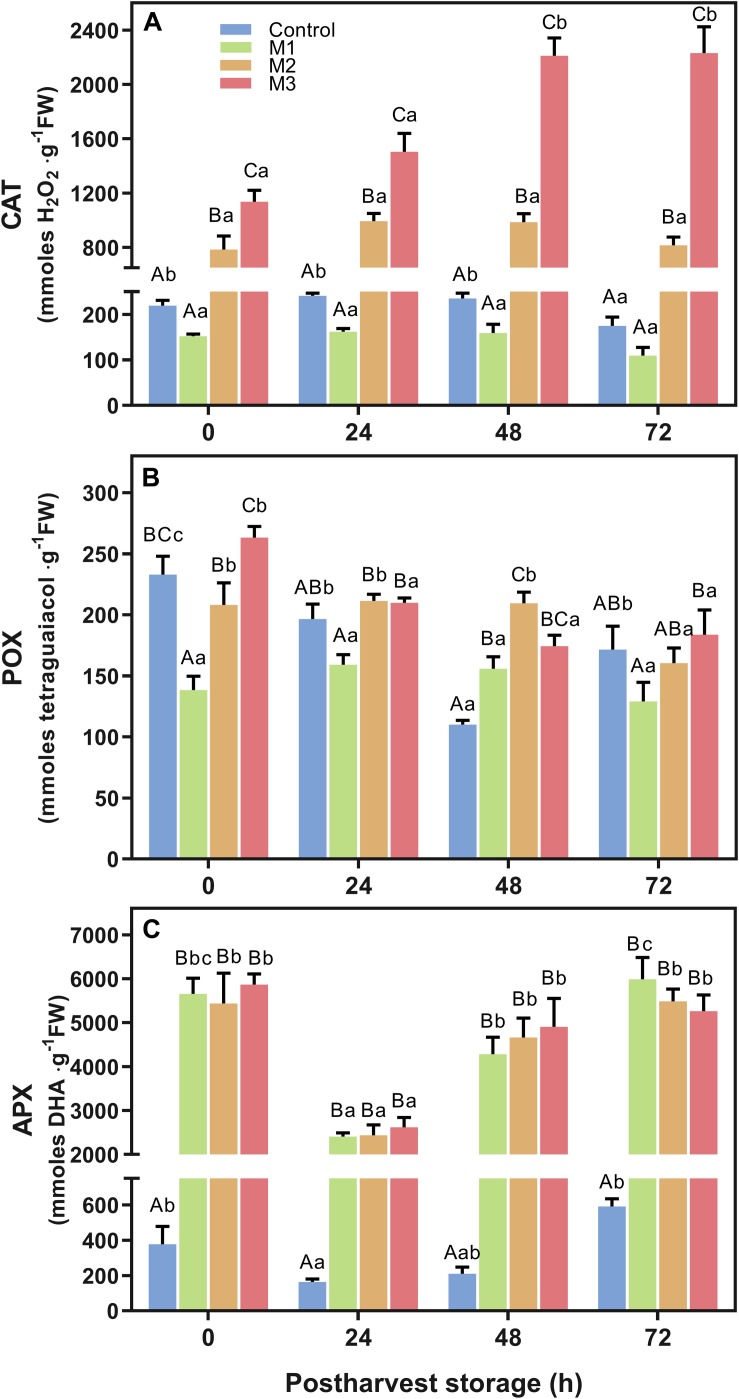
Changes in antioxidant-related enzymatic activities of preharvest methyl jasmonate (MeJA) treated strawberry (*Fragaria* × *ananassa* ‘Camarosa’) fruit at post-harvest. Effect of different MeJA treatments (M1, M2, and M3) or water (control) on strawberry antioxidant enzymatic activities of **(A)** catalase (CAT), **(B)** guaiacol peroxidase (POX), and **(C)** ascorbate peroxidase (APX) during post-harvest storage. Data represent mean ± SEM (*n* = 9). Differences between treatments were determined by ANOVA and Tukey test. Different capital letters indicate a significant difference between treatments within each post-harvest time point, and different lower-case letters indicate significant differences of each treatment between time points during post-harvest (*p* ≤ 0.05).

The effect of preharvest MeJA treatment on strawberry fruit antioxidant enzymatic activity of catalase (CAT), guaiacol peroxidase (POX), and ascorbate peroxidase (APX) is shown in Figure 6. Results revealed a significant increase in CAT activity in strawberry fruits (Figure 6A) depending on the number of preharvest MeJA applications (M2 and M3) compared to control fruits (*p* < 0.0001). Higher values were found on M3 treatment fruits at 0, 24, 48, and 72 h compared to control (5.2-, 6.1-, 9.4-, and 12.8-fold increases, respectively). Also, CAT activity increased two-fold during the post-harvest storage period between 0 and 72 h for M3 treatment. Along with it, M2 treatment remained approximately 4-fold higher than control at all time points during the post-harvest storage. Nevertheless, no differences were found between M1 and control fruits. Related to POX activity (Figure 6B), a similar response of M2 and M3 treatment at each time points were observed, but an unclear effect of preharvest MeJA applications was identified. No significant differences at 24 and 72 h between MeJA treatments and control were found, although a significant increase of POX activity on all MeJA treatments comparing with control was observed at 48 h. In contrast to CAT, we noticed a constant decrease in POX activity of M2 and M3 treatments during post-harvest storage time. Regarding APX activity (Figure 6C), even though no difference was observed between MeJA treatments, a higher increase was observed during post-harvest storage on all MeJA treatments, independent of the number of applications, compared to control. The average values of MeJA treatments showed a 15.0-, 15.3-, 22.3-, and 9.4-fold increases in relation to control at 0, 24, 48, and 72 h, respectively.

Finally, the complete dataset was analyzed by PCA (Supplementary Figure 3) to identify the major sources of variation responsible for the differences between treatments. The first two principal components (PC1 and PC2) explain 98% of the variability in the data set. In general, control and MeJA-treated fruits were grouped separately along the first and second axes, but no discrimination was observed based on the number of MeJA preharvest applications.

## Discussion

**FIGURE 7:**
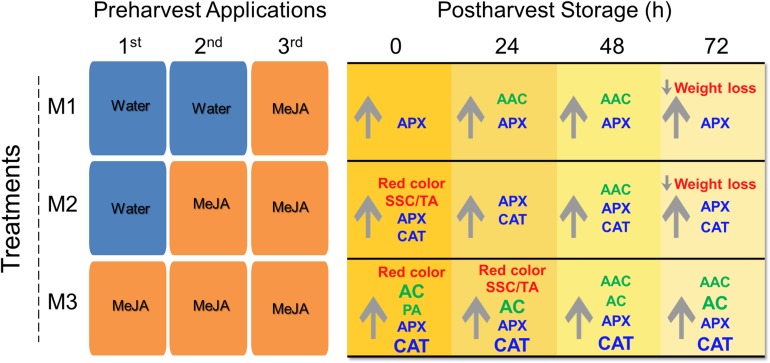
Post-harvest quality attributes of strawberry (*Fragaria* × *ananassa* ‘Camarosa’) fruit influenced by different preharvest methyl jasmonate (MeJA) treatments. Effect of different MeJA treatments (M1, M2, and M3) compared to control during post-harvest storage (0, 24, 48, 72 h). The same fruits were sprayed at flowering (M3), large green (M3 and M2), and 100% red receptacle (M3, M2, and M1) with 250 μmol L^–1^ MeJA. Upward and downward arrows indicate an increment and a decrease respect to control, respectively. In red font weight loss, red color (based on index a^∗^), SSC/TA ratio; in green font non-enzymatic antioxidants AAC, AC, PA; and in blue font enzymatic antioxidant activities (APX, CAT). Larger font size in CAT indicates significant higher values in M3 respect to M2 treatments. AAC, ascorbic acid content; AC, total anthocyanin content; APX, ascorbate peroxidase; CAT, catalase; PA, total proanthocyanidin content; SSC, soluble solids content; TA, titratable acidity. For experimental details (see section “Materials and Methods”).

In the present research, we report a significant impact of different preharvest MeJA treatments on different fruit quality parameters such as weight loss, soluble solids content/titratable acidity (SSC/TA) ratio, skin color, total anthocyanin (AC), total proanthocyanidin (PA) and ascorbic acid contents (AAC) accumulation, and antioxidant enzymatic activities of catalase (CAT) and ascorbate peroxidase (APX) during post-harvest storage (Figure 7). It is known that climatic conditions can influence fruit quality parameters in strawberry (Krüger et al., 2012) since we informed about climactic conditions during the field experiment (Supplementary Table 1). As far as we know, this is the first report about the effects of different MeJA preharvest applications on strawberry (*F.* × *ananassa* ‘Camarosa’) fruit quality parameters during post-harvest storage, and it can be useful to understand the mechanism involved in hormonal field application and its effect on the storability of soft fruits.

Concerning classical fruit quality parameters, we observed changes in weight loss, color, and SSC/TA ratio in preharvest MeJA-treated fruit during post-harvest. Previous reports suggest that MeJA applications during post-harvest of different fruits have an impact on declining the usual increment of fruit weight loss on treated blueberries (at 50 and 100 μmol L^–1^ MeJA) (Wang et al., 2019), apricots (at 200 μmol L^–1^ MeJA) (Ezzat et al., 2017), and strawberries (at 8 and 16 μmol L^–1^ MeJA) (Asghari and Hasanlooe, 2016; Ezzat et al., 2017; Wang et al., 2019). In the present study, the preharvest application of 250 μmol L^–1^ MeJA in large green and 100% red receptacle (M2 treatment) and in 100% red receptacle (M1 treatment) stages of strawberry (*F.* × *ananassa* ‘Camarosa’) fruit showed an effect on this attribute during post-harvest storage (Figure 1B) and could have a substantial impact on maintaining a critical commercial fruit quality attribute as weight.

In turn, fruit skin color is an essential trait for strawberry quality and has been associated with anthocyanin content and flavonoid composition (Fernández-Lara et al., 2015). In this sense, our results showed higher L^∗^ and a^∗^ values in MeJA-treated fruits (M2 and M3) than control at 0 and 24 h, indicating a brighter red color in ‘Camarosa’ fruits at the beginning of the post-harvest storage as a result of the preharvest treatment (Table 1). In other Rosaceae species such as apple (*Malus domestica* ‘Fuji’), preharvest application of MeJA also increased skin red color during fruit development (Rudell et al., 2005). Previous reports in strawberry, using exogenous MeJA applied in an *in vitro* ripening system, indicate a promoter role of this hormone in the acquisition of red coloration in fruit of commercial strawberry (*F.* × *ananassa* ‘Aromas’) (Delgado et al., 2018; Garrido-Bigotes et al., 2018), and Chilean strawberry (*F. chiloensis*) (Concha et al., 2013). All these antecedents suggest that MeJA could be used as a coloring promotor of strawberry, including field applications.

Another essential quality attribute of strawberry fruit is the flavor. In several fruits, an increase in the SSC and a concomitant reduction of the TA are observed during fleshy fruit ripening, which determine final fruit flavor and acceptance of fruit (Stevens et al., 1979; Cherian et al., 2014; Batista-Silva et al., 2018). Changes in the SSC/TA ratio by preharvest field MeJA applications have been reported in cultivars of blackberries (Wang et al., 2008), red raspberry (Wang and Zheng, 2005), and Chilean strawberry (Saavedra et al., 2016), but no effect has been observed in commercial strawberry (‘Tufts’ and ‘Cruz’ cultivars) at different MeJA concentrations (250, 500, and 1000 μmol L^–1^) applied at flowering (Yilmaz et al., 2003). Here, we found higher values of the SCC/TA ratio in M2 (at 0 and 48 h) and M3 (at 24 h) fruits compared with control (Table 1). In each case, the high SCC/TA ratio was explained by both, an increment on SSC and a diminution on TA of MeJA-treated fruits (Supplementary Table 1). In accordance with Yilmaz et al. (2003), which suggest that one MeJA application is not enough for modifying SSC/TA ratio (at 250, 500, or 1000 μmol L^–1^ MeJA), our results point out that at least two successive applications of 250 μmol L^–1^ MeJA since flowering (M3) or early fruit developmental (M2) stages are required on commercial strawberry to increase that parameter. Along with this, raspberry fruits treated with foliage-berry spray of 100 μmol L^–1^ MeJA at the early light pink stage with two consecutive sprayings, had higher SSC and lower TA and therefore a higher ratio of SSC/TA than control fruits or those treated with less MeJA concentrations (Wang and Zheng, 2005; Wang et al., 2008). Also, the highest fruit fructose and glucose contents and reduced fruit citric acid and malic acid contents were found in raspberry cultivars treated with MeJA (100 μmol L^–1^) (Wang and Zheng, 2005). The role of MeJA on the sugar content increase has been associated with the accumulation of anthocyanins and other phenolic compounds in the fruit, as has been shown in grapevine berries (Pirie and Mullins, 1976). However, the mechanism of the jasmonate-associated sugar accumulation should be uncovering in strawberry fruit.

In relation to metabolic changes suggested by changes in N (%) and C:N ratio (Figure 2), the MeJA applications assayed in the current study did not significantly modify nitrogen allocation and stoichiometry either in leaves or fruits of ‘Camarosa’ cultivar. In contrast, in other fruit species such as tomato, considering a biotic stress-related context, MeJA treatment induced a change in the allocation of resources (as C and N) (Gómez et al., 2010). As low nitrogen supply has been associated with an increase in anthocyanin levels in grape berry (Soubeyrand et al., 2014), we did not find an association with N content with the anthocyanin content in strawberry fruit. Therefore, our results suggest that MeJA applied to the whole strawberry plant during the fruit development period did not affect the allocation of N, and that the increment in fruit-related anthocyanin content (Figure 4A) could be promoted directly by MeJA application.

Regarding stress associated indicators, the analyses of δ^13^C and δ^15^N isotopes has been reported for the support that the application of MeJA could induce a more significant water use efficiency (WUE) and a differential allocation of nutrients, respectively, in fruits and leaves of different species as a defense mechanism to face both in biotic and abiotic stresses (Van Dam and Baldwin, 2001; Gómez et al., 2010). In this sense, the non-existence of differences in δ^13^C and δ^15^N between treatments both in leaves and fruits of MeJA-treated plants (Figures 3A–D), indicate that all plants were under the same irrigation and nitrogen fertilization management, and that MeJA did not interfere when the plant is in a non-stressed environment as has been previously shown in sugar beet and *Nicotiana attenuata* plants (Van Dam and Baldwin, 2001; Fugate et al., 2018). Moreover, we did not observed changes in malondialdehyde (MDA) levels in fruits treated with MeJA during post-harvest storage (Figure 3E), as an indicator of lipid peroxidation (Halliwell, 1987) and as has been reported in rice after MeJA application (Hung et al., 2006), which suggest the absence of oxidative stress at least in a high level. The lack of differences in our study suggests that these ranges of MeJA applications did not affect the level of lipid peroxidation in cell membranes at levels that cannot be compensated. Conversely, the MeJA applications could be associated with specific parameters of ripening and fruit quality. Nevertheless, additional research is needed to better understand the effect of field MeJA applications on the nutrient status (i.e., an experiment with differentiated N doses) and membrane integrity in strawberry.

The antioxidant systems in plants include antioxidant enzymes such as CAT, guaiacol peroxidase (POX), and APX, along with non-enzymatic antioxidants such as phenolic compounds, flavonoids, ascorbic acid among others, which are produced as secondary metabolites exerting various protective roles (Dixon and Paiva, 1995; Rice-Evans et al., 1996, 1997; Apel and Hirt, 2004). So, the content of these molecules and the activity of these enzymes can change by different growing conditions in several cultivars of *F.* × *ananassa* (Wang and Zheng, 2001; Wang et al., 2002). Phytohormones regulate many of these changes. Indeed, in different cultivars of blackberries, raspberries, black currants, plums, apples, and pomegranate fruits, MeJA treatment at preharvest increases the antioxidant capacity, total phenolic, and anthocyanin contents during post-harvest in a dependent concentration with a positive correlation between these values (Wang and Zheng, 2005; Wang et al., 2008; García-Pastor et al., 2020). In the present research, MeJA field applications on strawberry (*F.* × *ananassa* ‘Camarosa’) did not show a relation with those previously reported effects on the antioxidant capacity, total flavonoids content or total polyphenol content values up to 72 h of post-harvest storage. Similar results have been reported in post-harvest MeJA-treated strawberry (*F.* × *ananassa* ‘Coral’) since no differences in total phenolic content and antioxidant capacity was observed up to 5 days after treatment (de la Peña Moreno et al., 2010). Furthermore, we observed interesting changes in specific antioxidant-related mechanisms. It is known that MeJA application can increase anthocyanin accumulation in *F. chiloensis* and *F.* × *ananassa* ‘Camarosa’ and ‘Aromas’ cultivars in an *in vitro* ripening systems (Pérez et al., 1997; Concha et al., 2013; Delgado et al., 2018), because of a stimulatory effect on its biosynthesis through the activation of JA signaling that mean the upregulation of *FaMYC2* and *FaJAZ*s genes, increasing bioactive JA (jasmonoyl-isoleucine, JA-Ile) biosynthesis (Garrido-Bigotes et al., 2018), and consequently upregulating the regulatory (*FaMYB10*) and structural (*FaANS*, *FaUFGT*) genes related to anthocyanin biosynthesis pathway of strawberry (*F.* × *ananassa* ‘Aromas’) (Concha et al., 2013; Delgado et al., 2018). In the present research, we observed an accumulation of total anthocyanin content proportionally to the number of preharvest MeJA applications (Figure 4A) probably by means of the activation of JA signaling. Similarly, in *F.* × *ananassa* (‘Coral’) fruit treated with MeJA vapor at post-harvest, the anthocyanin content increased at 5 and 7 days post-harvest, being pelargonidin-3-glucoside, cyanidin-3-glucoside and pelargonidin-3-rutinoside the main augmented anthocyanins (de la Peña Moreno et al., 2010). In raspberry, preharvest MeJA treatment (100 μmol L^–1^) raised anthocyanins such as cyanidin 3-glucoside and cyanidin 3-rutinoside, respectively (Wang and Zheng, 2005). Besides, total proanthocyanidin content increase in M3 fruits but only at 0 h post-harvest (Figure 4B). Considering that the fruit color differences -darker red color or bright red color- in cultivars and selections of strawberry result from the content of anthocyanins (Hong and Wrolstad, 1990; Garzón and Wrolstad, 2002; da Silva et al., 2007; Kelebek and Selli, 2011; Song et al., 2015), our results suggest that, along with SCC/TA ratio, MeJA applications from flowering to ripe fruit stages have an essential impact on physiological characteristics during post-harvest storage, especially for the increase of anthocyanin levels during post-harvest and the coloring change (bright red color) at harvest of strawberry fruits.

Ascorbic acid is a non-enzymatic antioxidant that has an essential role in oxidative defense metabolism, maintaining cellular redox status, and scavenging over-production of reactive oxygen species (ROS) (Akram et al., 2017). In strawberry, ascorbic acid, along with anthocyanins, are responsible for between 55-70% (depending on the cultivar) of the total antioxidant capacity (Tulipani et al., 2008). Interestingly, we observed that the preharvest field MeJA applications (M1, M2, and M3) significantly enhanced the AAC during post-harvest (Figure 5). This finding was consistent with previous studies on strawberry (*F.* × *ananassa* ‘Selva’ and ‘Queen Elisa’ cultivars) (Lolaei et al., 2013), blueberry (Wang et al., 2019), and loquat fruit (Cai et al., 2011). Experiments on plant cell suspensions reports than MeJA can enhance the transcription of genes involved in the *de novo* biosynthesis of ascorbic acid (Wolucka et al., 2005). Besides to the non-enzymatic changes observed in the present study, preharvest MeJA field applications show an increase of antioxidant-related enzymatic activities of CAT and APX on strawberry (*F*. × *ananassa* ‘Camarosa’) fruits during post-harvest, being the M3 treatment that reached the highest levels of CAT activity at all post-harvest times (Figure 6A). Similar behavior was also reported on grape and blueberries, where post-harvest MeJA-treated fruits exhibited significantly higher APX and CAT activities during the storage (Modesti et al., 2018; Wang et al., 2019). Previously reported post-harvest treatment of 8 μmol L^–1^ MeJA on strawberry fruits (*F*. × *ananassa* ‘Sabrosa’) notified an increased CAT and POX activities (Asghari and Hasanlooe, 2016). Additionally, this effect is also shown on strawberry seedling leaves treated with 250 μmol L^–1^ MeJA (Faghih et al., 2017). In turn, preharvest MeJA treatments (100 μmol L^–1^) increase CAT, POX, and APX activities during lemon fruit development (Serna-Escolano et al., 2019). In general, we observed that an increase in CAT activity requires MeJA applications from flowering to ripe fruit stages, while APX activity can reach maximum values with just one application at ripe fruit stage (Figure 6C). Probably, the MeJA applications increase the levels of hydrogen peroxide (H_2_O_2_) in the strawberry plants, as has been early shown in several plant species (Orozco-Cardenas and Ryan, 1999). This increment could increase the activity of CAT and APX enzymes and thus inducing tolerance against oxidative stress, as has been demonstrated in tobacco plants (Gechev et al., 2002).

Finally, as the PCA analysis shows differentiation between control and MeJA-treated fruits (Supplementary Figure 3) and according to the summary scheme of our research (Figure 7) we conclude that MeJA treatment has significant beneficial effects on fruit quality at post-harvest storage, and three successive preharvest MeJA applications in different developmental strawberry (*F.* × *ananassa* ‘Camarosa’) fruit stages (flowering, large green, and 100% red) can markedly improve fruit quality and reinforce the antioxidant capacity that suggests a better status of the fruit to deal with oxidative stress during post-harvest. It is important to note that the results presented in the current research are related to the Camarosa cultivar, and these could be different using other varieties. All strawberry cultivars present fruits with a different composition of antioxidants (Diamanti et al., 2012a, b) that could interact with MeJA treatments in a different aspect as has been reported for black currant and raspberry cultivars (Flores and Ruiz del Castillo, 2015). By any means, preharvest MeJA applications could be incorporated into the integrated management programs for strawberry cultivation to get a better strawberry fruit quality for consumers. However, the use of an alternative source to analytical methyl jasmonate should be considered for cost reasons for strawberry growers (see Supplementary Table 3 for an estimate of the application cost per hectare based on the analytical compound used in the present study). Our interest in future research is to explore the MeJA-mediated regulation mechanism of the antioxidant-related enzymatic activity.

## Data Availability Statement

The datasets generated for this study are available on request to the corresponding author.

## Author Contributions

CF designed the research, supervised experiments, and acquired the funding. PZ, YC, OA-S, LF, and FA performed the experiments. PZ, OA-S, LF, FA, and CF analyzed the data. PZ, LF, and CF wrote the manuscript.

## Conflict of Interest

The authors declare that the research was conducted in the absence of any commercial or financial relationships that could be construed as a potential conflict of interest.
