# Supplementary material for: Methyl Jasmonate Applications From Flowering to Ripe Fruit Stages of Strawberry (Fragaria × ananassa ‘Camarosa’) Reinforce the Fruit Antioxidant Response at Post-harvest
Source: Front Plant Sci. 2020 May 8;11:538. doi: 10.3389/fpls.2020.00538 (PMC7225341; doi:10.3389/fpls.2020.00538)
Supplement: Supplementary file 1 [file Data_Sheet_1.docx]

Supplementary Material

# Supplementary Figures and Tables

## Supplementary Figures


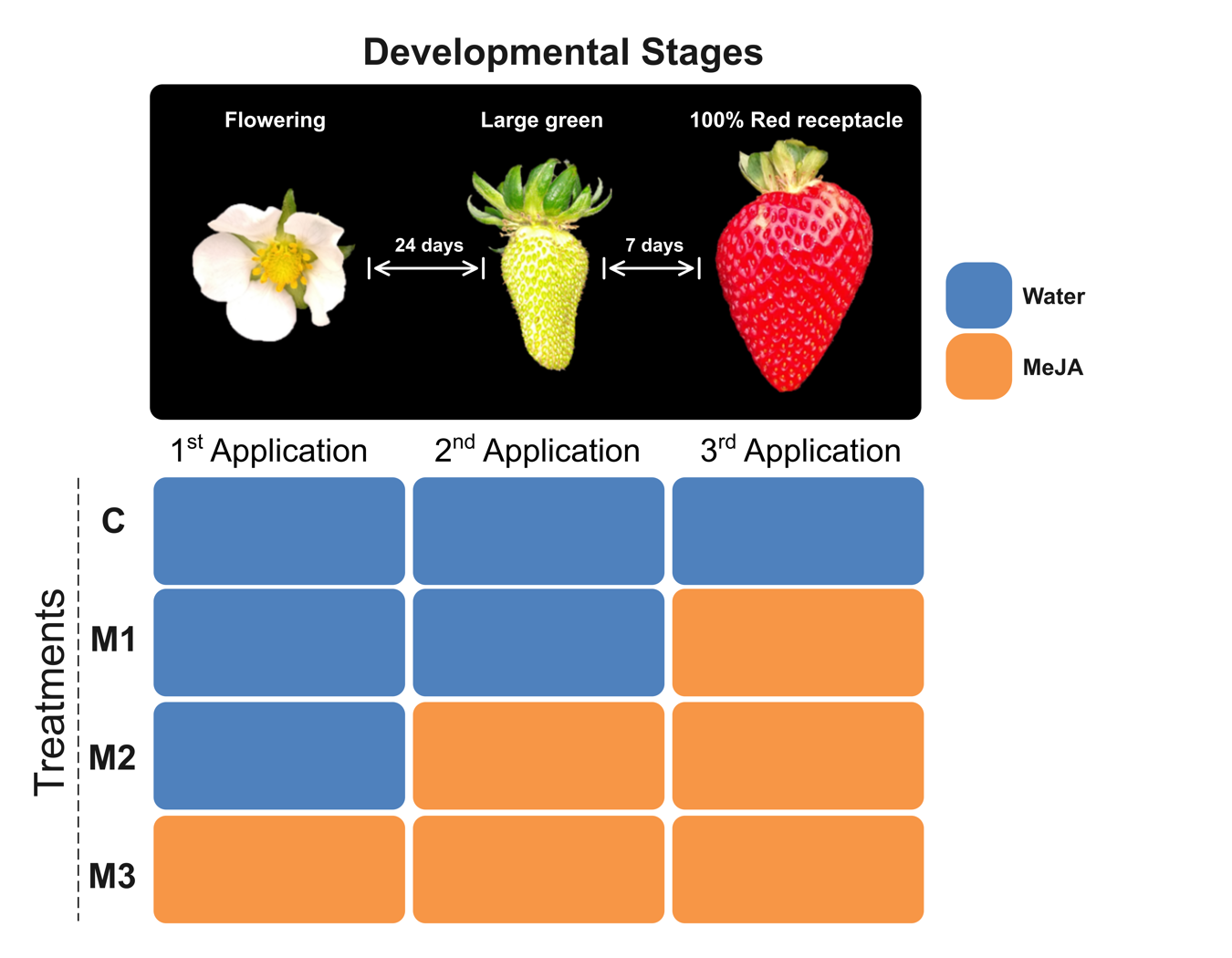


**Supplementary Figure 1. Field methyl jasmonate (MeJA) applications during strawberry** (*Fragaria* × *ananassa* ‘Camarosa’) **fruit development.** Three different sequential applications of 250 μmol L^-1^ MeJA (M1, M2, and M3) were performed through strawberry fruit development. The same fruits were sprayed at flowering (M3), large green (M3 and M2), and 100% red receptacle (M3, M2, and M1), according to each treatment. Distilled water was used as a control (C), and 0.05% (v/v) Tween-20 was adding as a surfactant in both solutions. About 100 plants distributed in three random plots were used per treatment. At each plot, 180 flowers were marked in the begin of this experiment. For experimental details, see the Materials and Methods section.

**
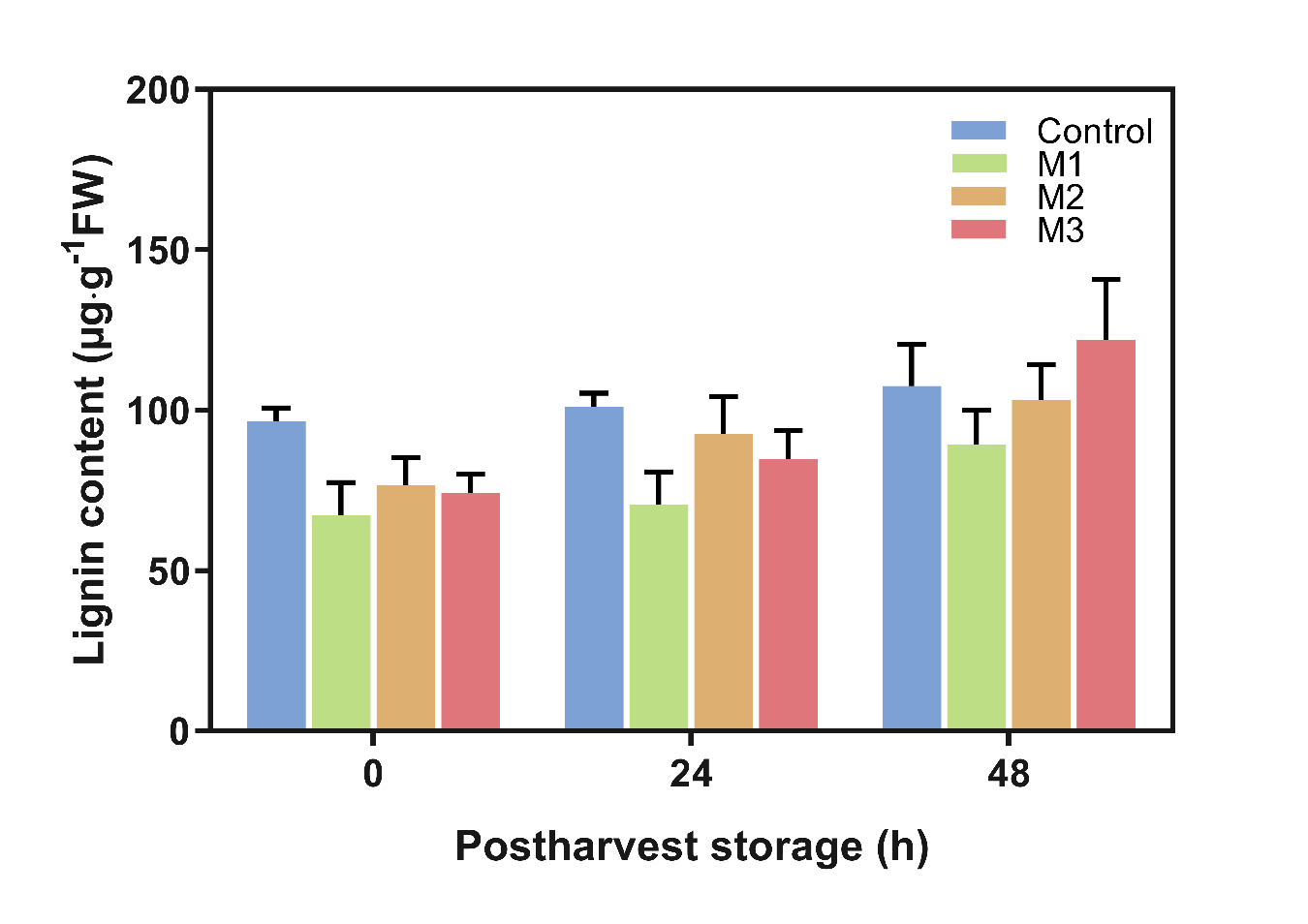
**

**Supplementary Figure 2.** Effect of different MeJA treatments (M1, M2, and M3) or water (control) on strawberry lignin content (µg per g of FW) during postharvest storage. Data represent mean ± SEM (n = 9). For experimental details, see the Materials and Methods section.

**
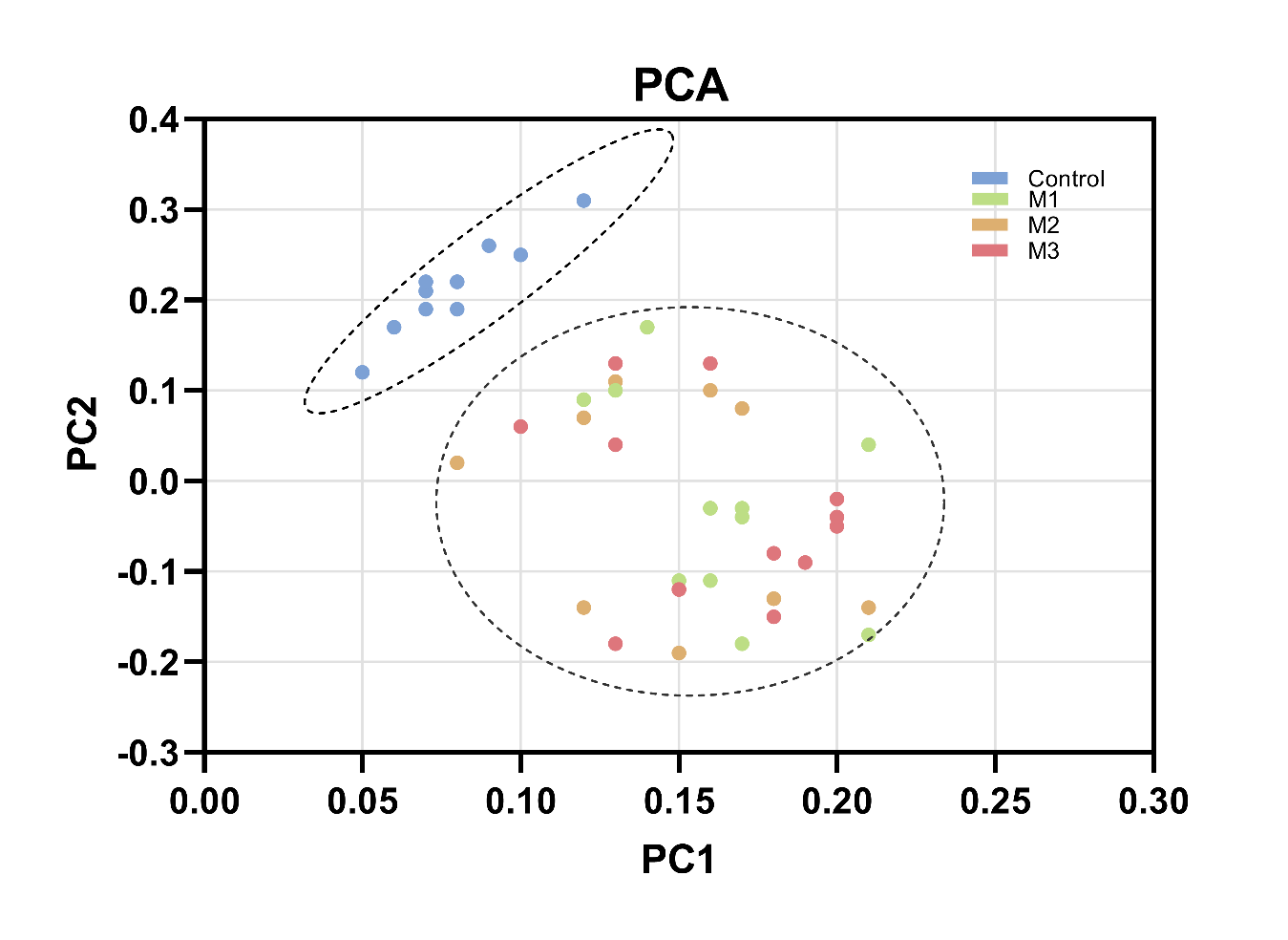
**

**Supplementary Figure 3**. Principal component analysis (PCA) of the relationship between the different MeJA treatments (M1, M2, and M3) or water (control) and the biochemical and physicochemical properties analyzed on strawberry fruits during postharvest storage in this research [weight loss; color index a*, b*, L*, Hue and Chroma; firmness; soluble solids content/titratable acidity ratio (SSC/TA); total flavonoid (TFC) and phenolic (TFC) contents; antioxidant capacity; lipid peroxidation; ascorbic acid content (AAC); total anthocyanin (AC) and proanthocyanidin (PA) contents; antioxidant enzymatic activities of catalase (CAT), guaiacol peroxidase (POX), and ascorbate peroxidase (APX)] according to dimension 1 and 2. The total variance explained by PCA was 98% (87% and 11% for PC1 and PC2, respectively). For experimental details, see the Materials and Methods section.

## Supplementary Tables

**Supplementary Table 1.** Climatic and soil temperature conditions on the site of study (Pelluhue, Maule Region, Chile) during preharvest field methyl jasmonate treatments in strawberry *Fragaria* × *ananassa* 'Camarosa'.

| **Parameter^1^** | **November 2016** | **December 2016** |
| --- | --- | --- |
| Cumulative precipitation (mm) | 422.3 | - |
| Relative humidity (%) | 78.1 | 76.5 |
| Average air temperature (°C) | 13.8 | 15.3 |
| Maximum air temperature (°C) | 26.7 | 24 |
| Minimum air temperature (°C) | 2.5 | 6.3 |
| Average soil temperature (°C) | 17.4 | 19.9 |
| Maximum soil temperature (°C) | 21.8 | 22.6 |
| Minimum soil temperature (°C) | 12.2 | 15.6 |

^1^Data were obtained from Center for Climate and Resilience Research (CR)^2^. DGF – FCFM, Universidad de Chile (2020). Explorador climático. <http://explorador.cr2.cl/> [Accessed March 3, 2020].

**Supplementary Table 2.** Changes in soluble solids content (SSC), titratable acidity (TA), and SSC/TA ratio during postharvest storage (0, 24, 48, and 72 h) of strawberry fruits treated with three different sequential applications of 250 μmol L^-1^ MeJA (M1, M2, and M3) or water (control) during preharvest.

| **Postharvest storage (h)** | **Treatments** | **SSC (g sucrose ∙100 g^−1^FW)** | **TA  (g CAE ∙100 g^−1^FW)** | **SSC/TA ratio** |
| --- | --- | --- | --- | --- |
| 0 | Control | 10.87 ± 1.77 ^Aa^ | 0.85 ± 0.11 ^ABa^ | 12.76 ± 1.70 ^Ac^ |
|  | M1 | 11.42 ± 1.31 ^Ab^ | 0.93 ± 0.08 ^Ba^ | 12.35 ± 1.41 ^Ab^ |
|  | M2 | 11.08 ± 1.58 ^Aa^ | 0.80 ± 0.11 ^Aa^ | 13.97 ± 1.30 ^Bb^ |
|  | M3 | 10.62 ± 1.44 ^Aa^ | 0.86 ± 0.16 ^ABa^ | 12.60 ± 2.66 ^Abc^ |
| 24 | Control | 10.17 ± 1.02 ^Aa^ | 1.04 ± 0.26 ^Bbc^ | 10.19 ± 2.26 ^Aab^ |
|  | M1 | 11.22 ± 0.97 ^BCb^ | 0.95 ± 0.05 ^ABa^ | 11.83 ± 1.18 ^ABb^ |
|  | M2 | 10.79 ± 1.24 ^ABa^ | 1.01 ± 0.14 ^ABb^ | 10.93 ± 1.92 ^Aa^ |
|  | M3 | 11.71 ± 1.44 ^Cb^ | 0.91 ± 0.17 ^Aa^ | 13.37 ± 3.02 ^Bc^ |
| 48 | Control | 10.54 ± 0.60 ^Aa^ | 0.96 ± 0.26 ^Bab^ | 11.40 ± 1.87 ^Ab^ |
|  | M1 | 11.01 ± 0.97 ^Ab^ | 0.89 ± 0.15 ^ABa^ | 12.90 ± 3.15 ^ABb^ |
|  | M2 | 10.94 ± 1.07 ^Aa^ | 0.84 ± 0.06 ^Aa^ | 13.14 ± 1.47 ^Bb^ |
|  | M3 | 10.54 ± 0.85 ^Aa^ | 0.91 ± 0.05 ^ABa^ | 11.62 ± 0.88 ^Aab^ |
| 72 | Control | 10.85 ± 1.66 ^ABa^ | 1.12 ± 0.17 ^Ac^ | 9.71 ± 0.52 ^Ba^ |
|  | M1 | 10.17 ± 0.79 ^Aa^ | 1.18 ± 0.13 ^Ab^ | 8.72 ± 0.87 ^Aa^ |
|  | M2 | 11.69 ± 1.65 ^Ba^ | 1.15 ± 0.15 ^Ac^ | 10.27 ± 1.38 ^Ba^ |
|  | M3 | 11.34 ± 1.04 ^Bab^ | 1.15 ± 0.26 ^Ab^ | 10.29 ± 2.34 ^Ba^ |

Data show mean values ± SD of three replicates (three fruits for each replicate). FW, fresh weight; CAE, Citric Acid Equivalent. For each parameter, different capital letters indicate a significant difference between treatments within each time point. Different lower-case letters indicate significant differences of each treatment between time points during postharvest (p ≤ 0.05). For experimental details, see the Materials and Methods section.

**Supplementary Table 3.** Estimate of the economic costs (USD) per hectare (ha) associated of preharvest methyl jasmonate (MeJA) treatments in strawberry (*Fragaria* × *ananassa* ‘Camarosa’) according to the number of applications of the present study.

| **Treatments** | **Total N° of applications** | **Total of 250 µmol L-1 MeJA required (L/ha)^1^** | **Cost (USD/ha)^2^** |
| --- | --- | --- | --- |
| M1 | 1 | 833.3 | 246.5 |
| M2 | 2 | 1666.6 | 493.0 |
| M3 | 3 | 2499.9 | 739.5 |

^1^ According to price information of USD 129 per 25 mL MeJA stock solution (CAS Number 39924-52-2) provided by Merck (Sigma-Aldrich, <https://www.sigmaaldrich.com/>, accessed March 3, 2020). Planting density of the MeJA-treated strawberry orchard was 50,000 plants/ha.
^2^ Other cost associated to the MeJA application are omitted.
